# The Genetic Effect of Copy Number Variations on the Risk of Type 2 Diabetes in a Korean Population

**DOI:** 10.1371/journal.pone.0019091

**Published:** 2011-04-22

**Authors:** Joon Seol Bae, Hyun Sub Cheong, Ji-Hong Kim, Byung Lae Park, Jeong-Hyun Kim, Tae Joon Park, Jason Yongha Kim, Charisse Flerida A. Pasaje, Jin Sol Lee, Yun-Ju Park, Miey Park, Chan Park, InSong Koh, Yeun-Jun Chung, Jong-Young Lee, Hyoung Doo Shin

**Affiliations:** 1 Laboratory of Genomic Diversity, Department of Life Science, Sogang University, Seoul, Republic of Korea; 2 Department of Genetic Epidemiology, SNP Genetics, Inc., Seoul, Republic of Korea; 3 Cancer Research Institute, Seoul National University College of Medicine, Seoul, Republic of Korea; 4 Integrated Research Center for Genome Polymorphism, School of Medicine, The Catholic University of Korea, Seoul, Republic of Korea; 5 Department of Hospital Pathology, Seoul St. Mary's Hospital, School of Medicine, The Catholic University of Korea, Seoul, Republic of Korea; 6 Center for Genome Sciences, National Institute of Health (NIH), Osong, Republic of Korea; 7 Center for Immunology and Pathology, National Institute of Health (NIH), Osong, Republic of Korea; 8 Department of Physiology, College of Medicine, Hanyang University, Seoul, Korea; South Texas Veterans Health Care System, United States of America

## Abstract

**Background:**

Unlike Caucasian populations, genetic factors contributing to the risk of type 2 diabetes mellitus (T2DM) are not well studied in Asian populations. In light of this, and the fact that copy number variation (CNV) is emerging as a new way to understand human genomic variation, the objective of this study was to identify type 2 diabetes–associated CNV in a Korean cohort.

**Methodology/Principal Findings:**

Using the Illumina HumanHap300 BeadChip (317,503 markers), genome-wide genotyping was performed to obtain signal and allelic intensities from 275 patients with type 2 diabetes mellitus (T2DM) and 496 nondiabetic subjects (Total *n* = 771). To increase the sensitivity of CNV identification, we incorporated multiple factors using PennCNV, a program that is based on the hidden Markov model (HMM). To assess the genetic effect of CNV on T2DM, a multivariate logistic regression model controlling for age and gender was used. We identified a total of 7,478 CNVs (average of 9.7 CNVs per individual) and 2,554 CNV regions (CNVRs; 164 common CNVRs for frequency>1%) in this study. Although we failed to demonstrate robust associations between CNVs and the risk of T2DM, our results revealed a putative association between several CNVRs including chr15:45994758–45999227 (*P* = 8.6E-04, *P^corr^* = 0.01) and the risk of T2DM. The identified CNVs in this study were validated using overlapping analysis with the Database of Genomic Variants (DGV; 71.7% overlap), and quantitative PCR (qPCR). The identified variations, which encompassed functional genes, were significantly enriched in the cellular part, in the membrane-bound organelle, in the development process, in cell communication, in signal transduction, and in biological regulation.

**Conclusion/Significance:**

We expect that the methods and findings in this study will contribute in particular to genome studies of Asian populations.

## Introduction

Type 2 diabetes mellitus (T2DM) is a serious metabolic disorder that is characterized by insulin resistance and hyperglycemia. This disease affects more than 170 million people worldwide, and is therefore a major health problem [1]. Since T2DM prevalence is higher in certain populations as suggested in twin and familial studies, scientists suspect that genetic factors play a significant role in the development of the disease [2]. The family aggregation analysis has proved to be a useful tool for genetic studies and to investigate predisposing factors of T2DM. Generally, the risk of developing T2DM is known to be approximately 3–4 times higher in the offspring of those with T2DM than in the offspring of those without a family history of diabetes. The lifetime risk of developing T2DM showed different ratios between offspring of one diabetic parent (40%) and of two diabetic parents (70%) to the risk of the disease [3], [4]. In order to identify genetic loci associated with T2DM, linkage, candidate, meta, and genome-wide association study (GWAS) analyses have been widely performed for various populations [5]. Among them, GWAS, which has allowed large-scale genotyping of hundreds of thousands of SNPs, is known as a powerful tool for scanning the disease-susceptible region at the whole-genome level [5]. Recently, the great development in microarray technology has increased the number of identified T2DM-susceptible gene loci using GWAS. To date, 25 common variations have been reported to be associated with T2DM through GWAS [6]. Compared to the abundant results of SNP GWAS, the few CNV GWAS's have been performed only in Caucasians, and none of the reports provided evidence of association with T2DM [7].

Ever since the duplication of the *Bar* gene and its contribution to the size of the *Bar* eye were identified in *Drosophila melanogaster* 70 years ago [8], large-scale genomic variations have usually been investigated using a microscope. In the past five years, comprehensive genomic variations, which cannot be visualized by microscope, have been revealed using high-density microarray and next-generation sequencing (NGS) platforms [9], [10]. CNV can contribute to disease susceptibility by influencing the gene expression level [11]. CNV has been revealed to be associated with complex human diseases including autism, inflammatory autoimmune disorders, lung cancer, osteoporosis, subarachnoid aneurysmal hemorrhage, sporadic amyotrophic lateral sclerosis, avellino corneal dystrophy, and schizophrenia [12], [13], [14], [15], [16], [17], [18], [19], [20], [21]. Recent studies have focused on how CNV in the human genome affects various inherited phenotypes, including disease susceptibility [22], [23]. Although the contribution of CNVs to various complex human diseases has been verified, studies on the association between the risk of T2DM and common CNVR in humans at the genome-wide level are still needed [7].

To identify disease-susceptible loci and putative CNV, PennCNV was performed using normalized signal intensity (log R ratio: LRR) and allelic intensity (B allele frequency: BAF) [17], [24], [25]. In addition, we speculate that the common CNVR is more suitable for use in investigating the risk factors of complex human diseases [14]. Therefore, this study used common CNVRs (CNV freq.>1%) that were obtained after samples with low quality were filtered following a strict criteria.

The current study demonstrates genome-wide individual CNVs identified using LRR and BAF, aggregated common CNVRs from identified individual CNVs, and results from a genome-wide association study with the risk of T2DM in a Korean population using a logistic regression model controlling for age and gender as covariates. We demonstrate our findings for three putative T2DM risk CNVRs through GWAS and their expected biological function.

## Materials and Methods

### Subjects and whole-genome SNP genotyping

A total of 275 unrelated patients with type 2 diabetes and 496 unrelated nondiabetic control subjects were recruited for this study. Diabetes was diagnosed based on the guidelines of the American Diabetes Association (ADA; 1997). Subjects with positive GAD antibodies were excluded. All subjects enrolled in this study were of Korean ethnicity. The study protocol was approved by the Institutional Review Board of the Clinical Research Institute at Korea National Institute of Health. Written informed consent was obtained from all subjects before drawing blood. Genome-wide SNP genotyping was performed using the Illumina HumanHap300 BeadChip containing 317,503 markers (Illumina, Inc., San Diego, CA, USA). Approximately 750 ng of genomic DNA extracted from the blood of each individual was used to genotype each sample. The assay procedure has been described in our previous study [12]. The overall SNP genotyping call rate of 99.91% in the current study indicated a high-quality data set. Power calculation of this GWAS was estimated by the QpowR program (https://www.msu.edu/~steibelj/JP_files/QpowR.html) using the R package. The power in this study was at 0.82 when sample size (n = 720), number of markers (n = 317,000), and default conditions were input into the Power calculator v1.0 of QpowR by parameters. The population admixture analysis was performed by the PCA analysis function in Golden HelixTree software (Golden Helix, Inc. Bozeman, MT, USA; http://www.goldenhelix.com) using ancestry informative markers (AIMs; n = 2,621). In order to compare the distribution from other populations, we used the genotypes from Japanese and Chinese cohorts. Figure S1 indicates that our GWAS samples showed homogeneity. Principal component analysis did not reveal any population stratification or population outliers (Figure S1).

### Identification of individual copy number variation

The signal intensity (LRR) and allelic intensity (BAF) ratios of all samples were exported using the Illumina BeadStudio software. Samples that did not satisfy the following criteria were excluded from the study: (i) call rate >99.0%; (ii) number of identified CNVs <100; and (iii) standard deviation of LRR <0.24, since samples with LRR SDs >0.24 are likely to have a low quality, resulting in a false positive CNV [26]. To identify individual CNVs, we incorporated multiple factors including log R ratio, B allele frequency, marker distance, and population frequency of the B allele using PennCNV [25], [27].

### Visual examination analysis and real-time quantitative PCR for CNV validation

**Figure 1:**
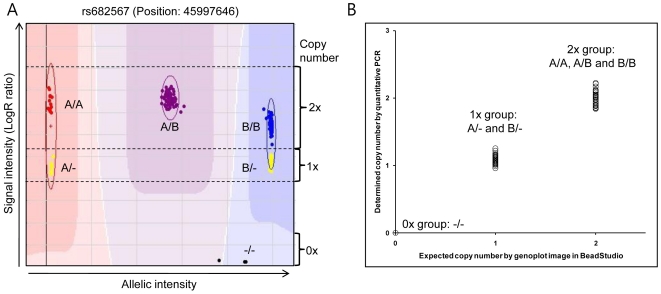
Copy number variation validation by qPCR around rs682567 within chr15:45994758–45999227. (A) Genoplot image of the identified deletions (marker name: rs682567). Genoplot image represents allelic intensity (X-axis) and signal intensity (Y-axis) of all samples. Two types of copy numbers are depicted as 2× and 1×. Individuals having hemizygous deletions (copy number: 1×) are clustered as two distinct groups (color: yellow). Samples having null copy numbers are displayed with a black dot at the bottom. (B) Validation by qPCR around the rs682567 within chr15: 45994758–45999227. The value of the X-axis (expected copy number) is estimated by the Illumina Genoplot image analysis. The Y-axis indicates the determined copy number by qPCR. The copy number value estimated through visual examination is matched with the quantitative measurement value by qPCR.

The genoplot image in Figure 1a can be beneficial in estimating copy number status of each subject. The image represents signal intensity (Y-axis) and allelic intensity (X-axis) for all subjects at one marker. Our previously reported result showed normal and abnormal genoplot images of whether the marker was located within the CNV or not [8]. If the marker was located within the CNV, six distinct cluster can be observed (CNV genotypes: A/A, A/B, B/B, A/-, B/-, and -/-). Two yellow-colored clusters in Figure 1a indicate subjects having a 1× copy number (CNV genotype: A/- and B/-, respectively). However, since this is an indirect estimation method by visual examination, we performed a direct quantitative PCR in selected subjects using the estimated copy number from the visual examination. In order to run qPCR, we designed a specific amplification primer set (forward primer sequence: 5′-GAAGAGGAGCAAGGCAAGGT-3′; reverse primer sequence: 5′-AGAATGACTCCTGTGGTCTTCACTA-3′) and TaqMan probe (5′-TACTGTGCTTACCTTCAAGACTAAC-3′) to validate the existence of the CNV within chr15:45994758–45999227, which is commonly associated with the risk of T2DM. Copy number determination analysis was performed using the ABI Prism 7900 sequence detection system. The *RNaseP* gene was co-amplified with the marker, which was then used as an internal standard. Amplification reactions (10 ul) were carried out using 10 ng of template DNA, 2× TaqMan® Universal Master Mix buffer (Applied Biosystems, Foster City, CA, USA), 900 nM of each primer, and 250 nM of each fluorogenic probe. Thermal cycling was initiated with 2 min incubation at 50°C, followed by a 10 min incubation at 95°C, and 40 cycles of 95°C 15 sec, 60°C 1 min. The first three replicate reactions were performed for similar primer pairs, and each copy number for an individual was calculated by Copy Caller v1.0 (Applied Biosystems) using the comparative C_T_ method.

### Logistic regression analysis and gene ontology analysis

CNVR is defined by merging overlapping CNVs. An input matrix is contained the status of samples in the each CNVR built from them. We generated input data for both loss (homozygous deletion = 0×; hemizygous deletion = 1×; normal copy number = 2×) and gain (normal copy number = 2×; duplication = 3×; amplification ≥4×). After constructing CNVRs from identified CNVs, the number of CNVs in the regions were counted in both case and control groups. Logistic regression analyses controlling for age (continuous value) and sex (male = 0 or female = 1) as covariates were used to calculate the *P*-values for case–control analysis. Multiple testing of CNVRs was carried out using the false discovery rate (FDR) method (frequency>1%). As long as it is a common multivariate logistic regression, this calculation can be easily done with any statistical tool such as the R package or SAS. Since there are many thousands of CNVRs, we built a java application for this repetitive calculation. This application also produces an input matrix from the CNV calls and the clinical information sheet, builds and trims different types of CNVRs, and calculates regression and FDR at a stroke. (The application will be announced and further described in a separate article.) In order to gain insights into the functional enrichment of CNVs, we performed gene ontology (GO) analysis using GOstat (http://gostat.wehi.edu.au) provided by Tim Beissbarth [28], [29].

## Results

In this study, we generated 7,478 individual CNVs and 2,554 CNVRs in a Korean population sample (n = 771) using the Illumina HumanHap300 BeadChip and PennCNV. The average number of CNVs per sample was 9.7, with an average length of 79.0 kb and a median size of 26.7 kb (Table S1). A total of 21 common CNVRs (frequency>5%) containing 21 genes, together with the rest of the regions, were found to overlap with previously reported CNVs in the Database of Genomic Variants (DGV) (Table 1). Furthermore, we were able to observe that 71.7% of the CNVs identified in this study matched those found in the DGV (Figure S2). However, all CNVs in the DGV overlapped with our results below 30%. We speculate that our results can explain only a small part of all human CNVs due to the sample size and ethnic differences. The size distribution of CNVs identified in this study is summarized in Figure S3, which shows that most were distributed within a range of 1–50 kb.

**Table 1:** Summary of common CNVRs (Freq.>5%).

CNV region	Length (kb)	No. of CNVs	CNV Frequency*	No. of genes	Gene	Overlapping with DGV**
chr18:64835114–64906488	71.4	169	0.219	1	*CCDC102B*	YES
chr19:20368239–20528316	160.1	145	0.188	2	*ZNF737,ZNF826*	YES
chr4:162050188–162154792	104.6	137	0.178	0		YES
chr14:43524894–43690442	165.5	99	0.128	0		YES
chr18:3559620–3561217	1.6	88	0.114	1	*DLGAP1*	YES
chr8:4905758–5947152	1041.4	84	0.109	0		YES
chr15:45994758–45999227	4.5	79	0.102	0		YES
chr11:5828407–5911385	83.0	66	0.086	2	*OR52E4,OR52E8*	YES
chr21:16693940–16716168	22.2	57	0.074	1	*C21orf34*	YES
chr8:15447669–15471819	24.2	54	0.070	1	*TUSC3*	YES
chr22:20352005–21702142	1350.1	52	0.067	13	*GGTLC2,LOC648691,LOC96610,MAPK1,POM121L1P,PPIL2,PPM1F,PRAME,TOP3B,VPREB1,YPEL1,ZNF280A,ZNF280B*	YES
chr4:153203765–153212191	8.4	50	0.065	0		YES
chr3:65166887–65187636	20.7	48	0.062	0		YES
chr6:31463297–31572718	109.4	48	0.062	3	*HCG26,HCP5,MICA*	YES
chr12:130289496–130380887	91.4	47	0.061	0		YES
chr9:11743695–12194748	451.1	46	0.060	0		YES
chr10:90934639–90935788	1.1	44	0.057	0		YES
chr12:7861988–8017012	155.0	44	0.057	2	*SLC2A14,SLC2A3*	YES
chr8:145028388–145358471	330.1	42	0.054	13	*C8orf30A,CYC1,EXOSC4,GPAA1,GRINA,HEATR7A,KIAA1875,MAF1,OPLAH,PARP10,PLEC1,SHARPIN,SPATC1*	YES
chr5:32137157–32205304	68.1	40	0.052	2	*GOLPH3,PDZD2*	YES
chr5:41251228–41270765	19.5	40	0.052	1	*C6*	YES

*The CNV frequencies were obtained by number of total identified CNVs/number of total subjects.

**Database of genomic variants (http://projects.tcag.ca/variation).

To identify new genetic risk factors that may contribute to the development of T2DM, we performed a genome-wide association study (GWAS) of CNVs in a total of 771 Korean ethnic individuals, including T2DM patients (n = 275) and control subjects (n = 496). Logistic regression analyses controlling for age and gender as covariates were performed to carry out the GWAS of 164 common CNVRs (frequency>1%) with T2DM. Gene ontology (GO) analysis can provide insights into the functional enrichment of identified CNVs. After we extracted genes covering the CNVs identified in this study, we performed GO analysis. As a result, we found that genes significantly enriched in the identified CNVs include cell part, organelle, development process, biological regulation, and signal transduction (Table S2).

**Figure 2:**
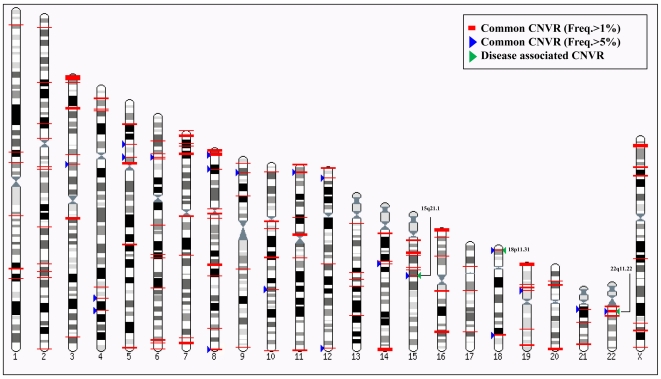
Map of identified common copy number variation and T2DM-associated regions. This figure shows common CNVRs (freq.>1%, red-colored rectangle) and highly common CNVRs (freq.>5%, blue colored triangle) from CNVs identified in this study. Putative diseases associated CNVRs are marked by a green-colored triangle. The location of each CNVR was displayed by the Karyoview of Ensembl (http://apr2006.archive.ensembl.org/Homo_sapiens/karyoview) according to our previous method [41].

Three novel candidate T2DM loci, chr15:45994758–45999227 (15q21.1, *P* = 8.6E-4, *P^corr^* = 0.01), chr22:20722473–21702142 (22q11.22, *P* = 0.009, *P^corr^* = 0.06), and chr18:3559620–3561217 (18p11.31, *P* = 0.01, *P^corr^* = 0.06), were found to be significantly associated with the risk of T2DM in a Korean population (Table 2, Figure 2). One CNVR (chr15:45994758–45999227) was associated with susceptibility to T2DM even after FDR corrections for multiple testing (OR = 2.6, 95% CI = 1.5–4.5, *P* = 0.01, Table 2). Figure S4 shows the positional and probe information from the DGV genome browser around 15q21.1 (chr15:45994758–45999227). This target CNV covers three consecutive markers (rs549368, rs682567, and rs2965306). Two nearby genes, *SEMA6D* and *SLC24A5*, were distributed in 141.0 kb and 201.2 kb distances, respectively. Furthermore, two previously reported CNVs (variation_10483 and variation_38328) on DGV overlapped with the CNVs in this study. In order to validate the quantitative change of copy number for the identified T2DM-susceptible CNVR, we selected rs682567 within chr15:45994758–45999227 and performed quantitative PCR (qPCR) (Figure 1). Results of visualization from the genoplot image showed a copy number change between patients and controls, indicating that chr15:45994758–45999227 might be a T2DM-susceptible region.

**Table 2:** Logistic regression analysis of identified CNVRs with risk of type 2 diabetes in Korean subjects (n = 771).

CNV region*	Length (kb)	Cytoband	Genes (Nearby genes)	Type	Case (Freq.**)	Control (Freq.**)	OR (95% CI)	*P*	*P^corr^* †	DGV
chr15:45994758–45999227	4.5	15q21.1	(*SEMA6D, SLC24A5*)	Deletion	39(7.86%)	39(14.18%)	2.6 (1.5–4.5)	**8.6E-04**	**0.01**	Overlapped
chr22:20722473–21702142	979.7	22q11.22	*GGTLC2,LOC648691,LOC96610,POM121L1P,PRAME,VPREB1,ZNF280A,ZNF280B*	Deletion	27(5.44%)	4(1.45%)	0.2 (0.7–0.1)	**0.009**	0.06	Overlapped
chr18:3559620–3561217	1.6	18p11.31	*DLGAP1*	Deletion	45(9.07%)	43(15.64%)	2.0 (1.2–3.4)	**0.01**	0.06	Overlapped

Abbreviations: CNV (copy number variation), OR (odds ratios), CI (confidential interval), *P^corr^* (corrected *P* value), DGV (Database of Genomic Variants).

*The version of human reference genome: NCBI build 36/hg18.

**The frequency was obtained by number of identified CNVs/number of case subjects in case group and number of identified CNVs/number of control subjects in control group.

† The false discovery rate (FDR) method used to perform a multiple testing of copy number variation regions (CNVRs) (Freq.>1%).

Bold values indicate the case of *P*<0.05.

## Discussion

Type 2 diabetes mellitus (T2DM) is a metabolic disorder characterized by high glucose level in the context of insulin resistance and relative insulin deficiency [30]. Although the distinctive causes and factors of diabetes have not been clarified, the development of T2DM has been known to be influenced by genetic and environmental factors. Currently, many studies are attempting to discover genes causing T2DM, and remarkable numbers of susceptible genes have been found. In particular, exponential progress in the development of a high-density chip platform has led to robust studies with GWAS analysis, a method that uses a chip with hundreds of thousands of markers. As a result, genes such as *CDKAL1*, *SLC30A8*, *HHEX*, *EXT2*, *IGF2BP2*, *CDKN2B*, *LOC387761*, *FTO*, *KCNQ1*, *TCF7L2*, and *BCL11A* have been reported to be susceptible genes for T2DM [6], [31], [32]. However, most of the current research only focuses on SNPs as genetic markers, whereas T2DM studies using CNV as a newly discovered genetic marker are relatively rare.

Recently, Craddock et al. reported that the *TSPAN8* gene may be a putatively associated candidate gene in T2DM, using large samples and a comparative genomic hybridization (CGH) array (*P* = 3.9E-5) [7]. However, they failed to identify CNVs that greatly contribute to the risk of T2DM, a conclusion that is similar to the results in this study. In addition, Shtir et al. conducted a study on the association between CNVs and T2DM in 194 Caucasian patients. However, they were able to show few evidences of such association [33]. Another report, from Jeon et al., has shown that CNV in the leptin receptor gene is associated with T2DM [34]. Our study confirmed the existence of CNV in the leptin receptor gene by examining intensity change on a 50K chip and conducting qPCR on this gene, which could be a good reference model for future target gene approach studies of CNVs. However, Jeon et al's study also used a low-density chip (50K), so it would have been hard to discover T2DM-susceptible genes through GWAS. Instead, we used the PennCNV software, a widely used technology for CNV research [12], [14], [17], [25], [27], [35], [36], [37], [38], [39], [40], [41], with samples that passed the strict quality control step. To perform an accurate association analysis, we also utilized signal intensity, allelic intensity, and population frequency of the B allele for reliable CNV identification [25], and logistic regression analysis on common CNVRs was carried out, controlling for age and gender as covariates.

To identify more reliable CNVs, we used the Illumina HumanHap300 BeadChip, which contains 317,503 markers. We found 7,478 individual CNVs, 2,554 CNVRs, and 164 common CNVRs (frequency>1%) in a Korean population (n = 771). We also compared our results on the individual CNVs with those found in the DGV database. Our findings revealed that the CNVs identified in this study were in concordance with the DGV database at 71.7%, an indication of high-quality CNVs. The CNVs (28.3%) that did not match the data in the DGV may have been due to ethnic or chip platform differences. In addition, results from gene ontology (GO) analysis showed that CNVs identified in this study were enriched in the pathways of cell parts, protein binding, developmental processes, biological regulations, and cell communication in the GO category (Table S2).

To assess disease-susceptible loci for risk of T2DM, genome-wide CNV association analysis using a logistic regression model was performed. In this study, we found that three new CNVRs (chr15:45994758–45999227, chr22:20722473–21702142, and chr18:3559620–3561217) were significantly associated with the risk of T2DM. In particular, the chr15:45994758–45999227 region showed the strongest association signal that was retained even after multiple corrections (*P^corr^* = 0.01). Interestingly, the *SEMA6D* (sema domain, transmembrane domain [TM], cytoplasmic domain [semaphoring] 6D) and *SLC24A5* (solute carrier family 24, member 5) genes are located within 200 kb from this CNVR (Figure S4). Although the association signals for two other new CNVRs (chr22:20722473–21702142 and chr18:3559620–3561217) disappeared after multiple corrections (*P^corr^* = 0.06 and *P^corr^* = 0.06, respectively), those regions covered nine T2DM candidate genes including gamma-glutamyltransferase light chain 2 (*GGTLC2*); *LOC648691*; *LOC96610*; POM121 membrane glycoprotein-like 1, pseudogene (*POM121L1P*); preferentially expressed antigen in melanoma (*PRAME*); pre-B lymphocyte 1 (*VPREB1*); zinc finger protein 280A (*ZNF280A*); zinc finger protein 280B (*ZNF280B*); discs, and a large [Drosophila] homolog-associated protein 1 (*DLGAP1*). Most of the identified candidate genes were related to immune responses and transcriptional regulations, although the molecular function and the relationship with the onset of T2DM are still unknown. Therefore, further studies are required to clarify the role of these genes in an individual's susceptibility to T2DM.

In conclusion, we found several CNVRs that were significantly associated with the risk of T2DM in a Korean population. Although our findings provide preliminary results on the relationship between CNV and T2DM, future replication analyses in larger independent cohorts from various ethnic populations, and functional studies exploring the underlying mechanisms of association between the susceptible loci discovered in this study and T2DM, are also necessary. In addition, rare CNVs have been recently considered as important variations in association analyses between rare CNV and diseases. Therefore, we suggest that rare CNVs from family samples or larger-scale samples can provide more advanced insights into T2DM genetics. We believe that the findings and methods in this study will serve as important resources in future human genome research.

## Supporting Information

Figure S1 **PC plot.** First and second principal component of our samples (red: cases; blue: controls) together with the Japanese (green triangles) and Chinese (green squares) HapMap samples using a set of East Asian ancestry informative markers.
(PDF)

Figure S2 **Overlapping result of identified CNVs in this study with DGV database.**
(PDF)

Figure S3 **Size distribution of identified copy number variations and aggregated copy number variation regions in a Korean population (n = 771).**
(PDF)

Figure S4 **Region around chr15:45994758–45999227 containing nearby genes and previously reported CNVs in the region.** The figure is developed from the DGV genome browser. Markers of this study are indicated by a diamond, and the previously reported CNVs in the DGV are noted by a filled block.
(PDF)

Table S1 **Summary of identified CNV in this study.**
(DOC)

Table S2 **Gene ontology categories significantly overrepresented in identified CNV.**
(DOC)
