# Supplementary figures and images for: The Genetic Effect of Copy Number Variations on the Risk of Type 2 Diabetes in a Korean Population
Source: PLoS One. 2011 Apr 22;6(4):e19091. doi: 10.1371/journal.pone.0019091 (PMC3081314; doi:10.1371/journal.pone.0019091)

Figure S1

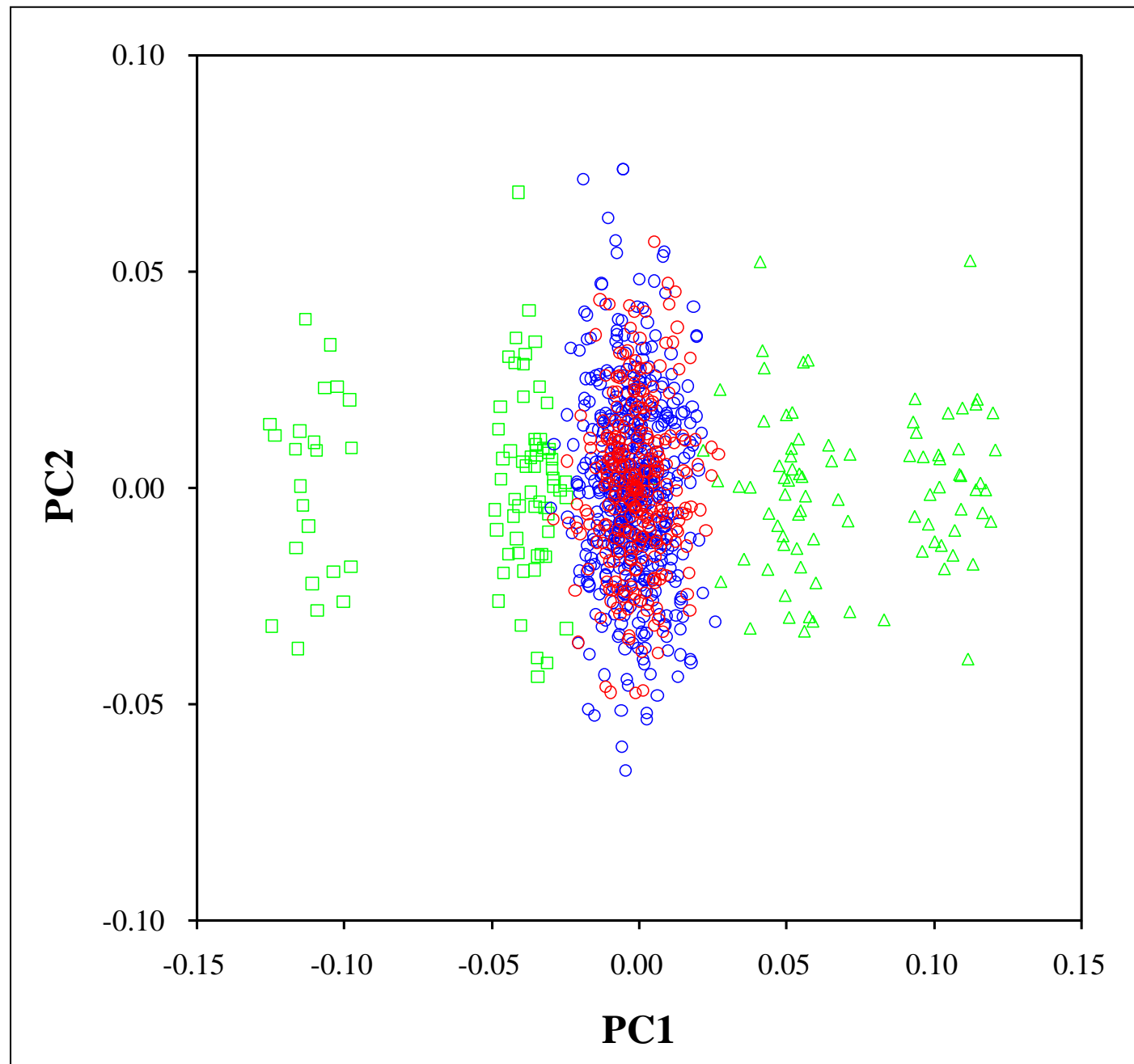

Supplement: Figure S1 — PC plot. First and second principal component of our samples (red: cases; blue: controls) together with the Japanese (green triangles) and Chinese (green squares) HapMap samples using a set of East Asian ancestry informative markers. (PDF) [file pone.0019091.s001.pdf]

Figure S2

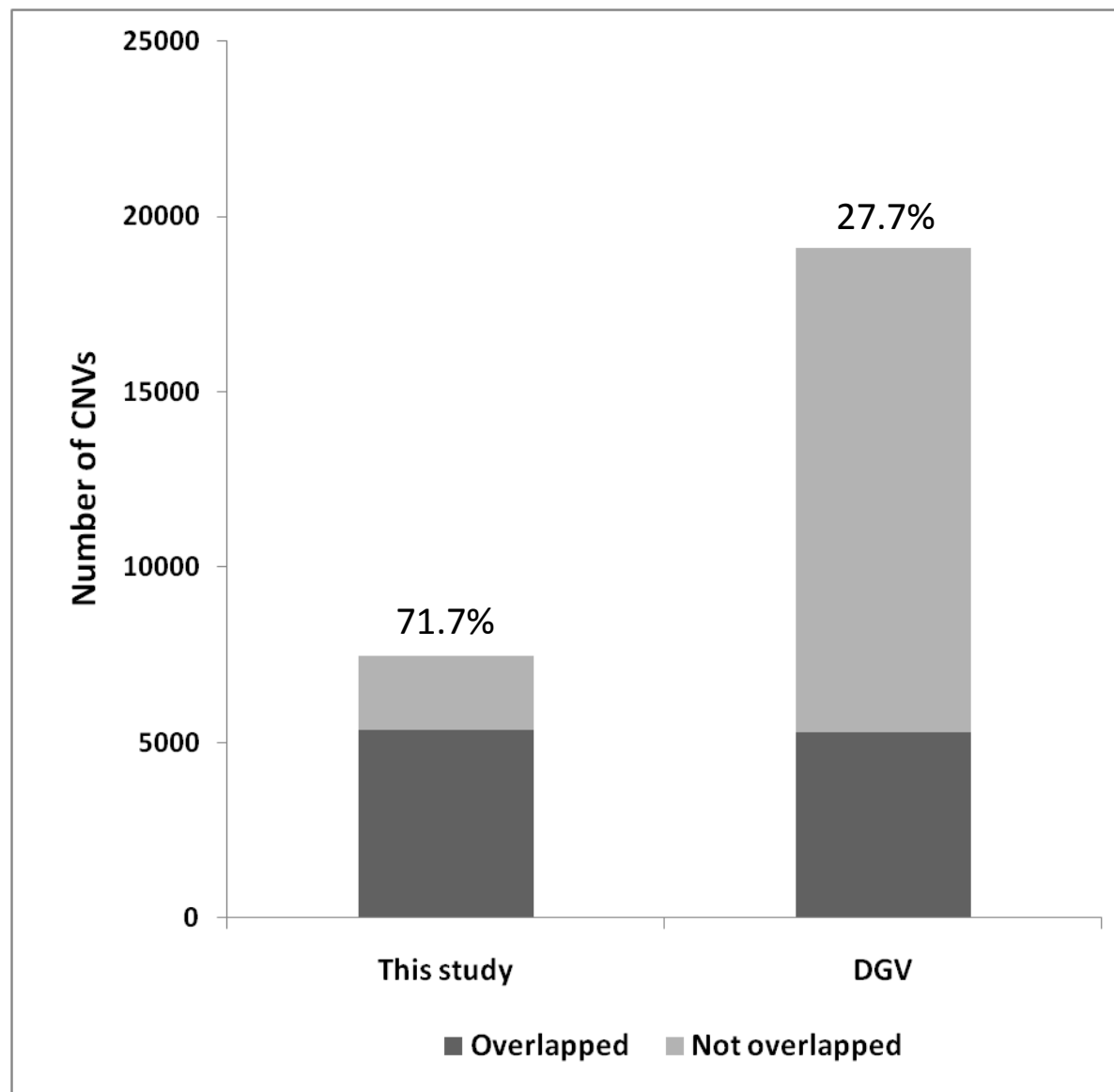

Supplement: Figure S2 — Overlapping result of identified CNVs in this study with DGV database. (PDF) [file pone.0019091.s002.pdf]

Figure S3

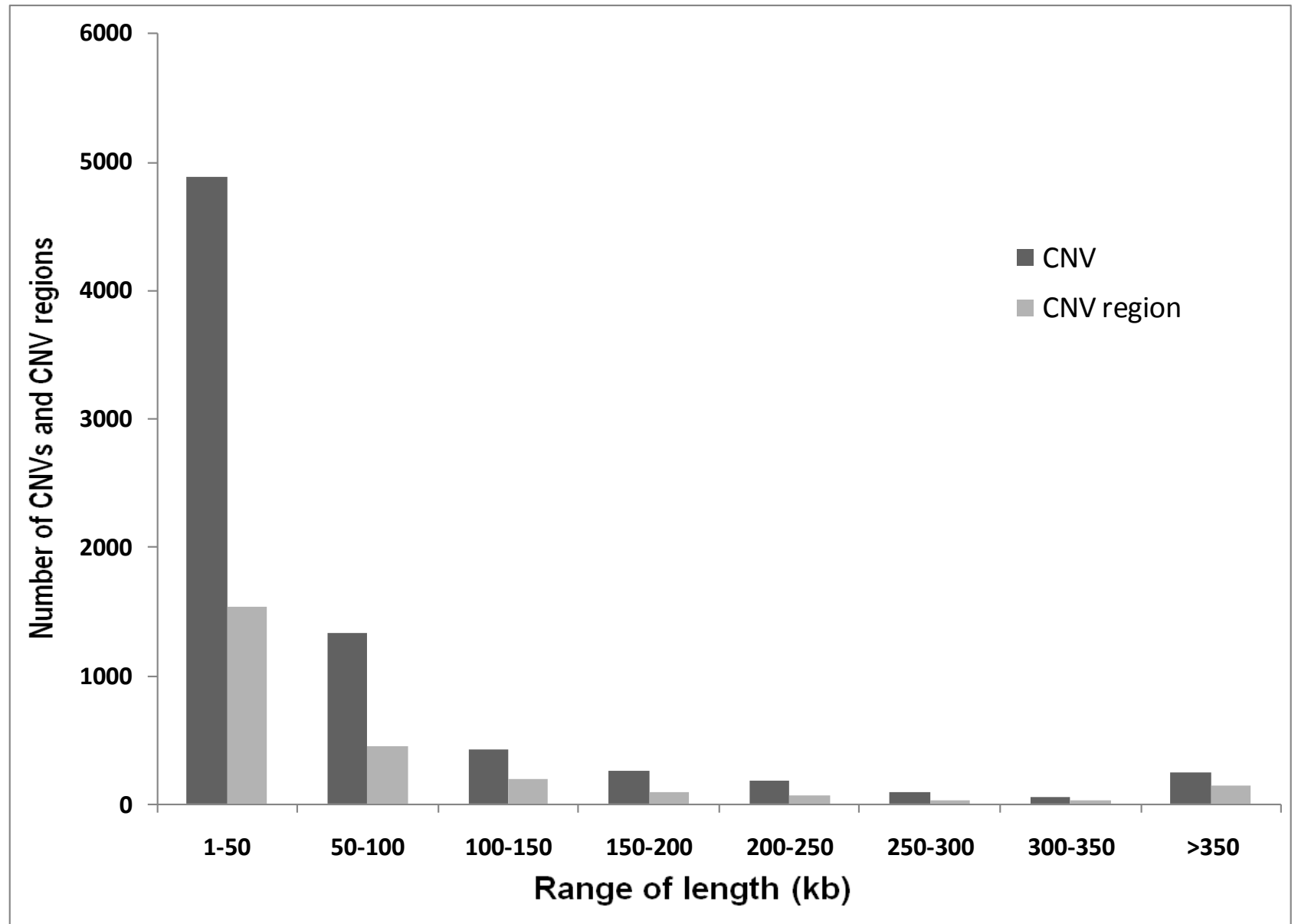

Supplement: Figure S3 — Size distribution of identified copy number variations and aggregated copy number variation regions in a Korean population (n = 771). (PDF) [file pone.0019091.s003.pdf]

Figure S4

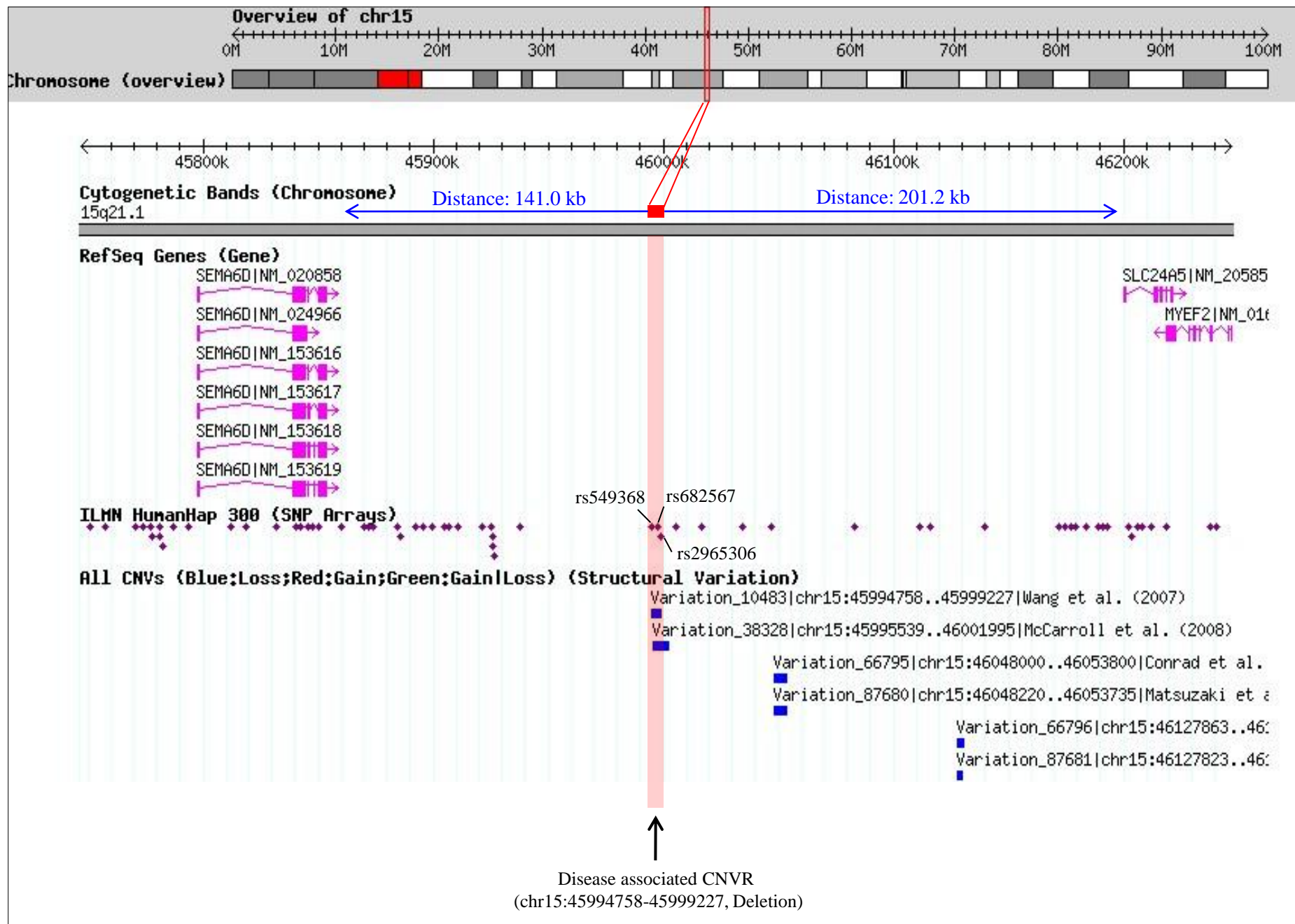

Supplement: Figure S4 — Region around chr15:45994758–45999227 containing nearby genes and previously reported CNVs in the region. The figure is developed from the DGV genome browser. Markers of this study are indicated by a diamond, and the previously reported CNVs in the DGV are noted by a filled block. (PDF) [file pone.0019091.s004.pdf]
