# Supplementary material for: The Genetic Effect of Copy Number Variations on the Risk of Type 2 Diabetes in a Korean Population
Source: PLoS One. 2011 Apr 22;6(4):e19091. doi: 10.1371/journal.pone.0019091 (PMC3081314; doi:10.1371/journal.pone.0019091)
Supplement: Table S1 — Summary of identified CNV in this study. (DOC) [file pone.0019091.s005.doc]

| Table S1. Summary of identified CNV in this study | | | |
| --- | --- | --- | --- |
| Parameters | Case  (n=275) | Control (n=496) | Total (n=771) |
| Total number | 3,482 | 3,996 | 7,478 |
| Avg. number of CNVs per sample^*^ | 12.7 | 8.1 | 9.7 |
| Avg. size of CNVs (kb) | 66.8 | 89.6 | 79.0 |
| Median size of CNVs (kb) | 21.8 | 30.3 | 26.7 |
| Number of gain | 1,280 | 1,540 | 2,820 |
| Number of loss | 2,202 | 2,456 | 4,658 |
| Ratio (Loss/Gain) | 1.7 | 1.6 | 1.7 |

^*^This value was calculated by each formula ([Identified CNVs in case group/total number of sample in case group] and [Identified CNVs in control group/total number of sample in control group]). The difference between case and control group means that the genomic variation is frequent in cases rather than controls. Result reveals a significant association between two groups (*P* = 0.0004).
