# Supplementary material for: The Genetic Effect of Copy Number Variations on the Risk of Type 2 Diabetes in a Korean Population
Source: PLoS One. 2011 Apr 22;6(4):e19091. doi: 10.1371/journal.pone.0019091 (PMC3081314; doi:10.1371/journal.pone.0019091)
Supplement: Table S2 — Gene ontology categories significantly overrepresented in identified CNV. (DOC) [file pone.0019091.s006.doc]

| Table S2. Gene ontology categories significantly overrepresented in identified CNV | | | | |
| --- | --- | --- | --- | --- |
|  |  |  |  |  |
| GO id | Gene ontology | Count | Total | *P*-value^*^ |
| GO:0044464 | cell part | 1621 | 21746 | 7.73E-52 |
| GO:0005515 | protein binding | 835 | 9005 | 2.74E-51 |
| GO:0044425 | membrane part | 700 | 7726 | 2.15E-36 |
| GO:0005886 | plasma membrane | 405 | 3816 | 3.88E-35 |
| GO:0005737 | cytoplasm | 674 | 7482 | 7.09E-34 |
| GO:0044424 | intracellular part | 1038 | 12958 | 9.75E-34 |
| GO:0016020 | membrane | 828 | 9747 | 1.37E-33 |
| GO:0043227 | membrane-bound organelle | 745 | 8827 | 6.36E-28 |
| GO:0043231 | intracellular membrane-bound organelle | 744 | 8824 | 9.16E-28 |
| GO:0031224 | intrinsic to membrane | 598 | 6732 | 2.62E-27 |
| GO:0016021 | integral to membrane | 594 | 6700 | 7.50E-27 |
| GO:0032501 | multicellular organismal process | 379 | 3822 | 1.46E-25 |
| GO:0043229 | intracellular organelle | 854 | 10763 | 1.67E-23 |
| GO:0043226 | organelle | 854 | 10768 | 1.84E-23 |
| GO:0005622 | intracellular | 1111 | 14906 | 7.90E-23 |
| GO:0032502 | developmental process | 334 | 3347 | 9.52E-23 |
| GO:0044459 | plasma membrane part | 248 | 2285 | 3.44E-22 |
| GO:0065007 | biological regulation | 575 | 6731 | 2.25E-21 |
| GO:0007154 | cell communication | 490 | 5560 | 1.28E-20 |
| GO:0048856 | anatomical structure development | 217 | 2005 | 5.37E-19 |
| GO:0007275 | multicellular organismal development | 237 | 2299 | 1.44E-17 |
| GO:0050789 | regulation of biological process | 516 | 6140 | 5.10E-17 |
| GO:0007165 | signal transduction | 443 | 5142 | 3.36E-16 |
| GO:0048731 | system development | 176 | 1605 | 7.84E-16 |
| GO:0031226 | intrinsic to plasma membrane | 152 | 1346 | 6.73E-15 |
| GO:0005624 | membrane fraction | 91 | 683 | 5.39E-14 |
| GO:0000267 | cell fraction | 111 | 902 | 7.15E-14 |
| GO:0005887 | integral to plasma membrane | 148 | 1330 | 8.22E-14 |
| GO:0048503 | GPI anchor binding | 29 | 126 | 3.10E-13 |
| GO:0007399 | nervous system development | 92 | 716 | 7.35E-13 |
| GO:0030054 | cell junction | 64 | 429 | 7.68E-13 |
| GO:0016043 | cellular component organization and biogenesis | 293 | 3277 | 3.93E-12 |
| GO:0048519 | negative regulation of biological process | 131 | 1182 | 5.72E-12 |
| GO:0050794 | regulation of cellular process | 460 | 5704 | 1.69E-11 |
| GO:0044444 | cytoplasmic part | 382 | 4592 | 3.74E-11 |
| GO:0048523 | negative regulation of cellular process | 125 | 1137 | 4.38E-11 |
| GO:0005634 | nucleus | 480 | 6058 | 8.97E-11 |
| GO:0031175 | neurite development | 28 | 135 | 1.33E-10 |
| GO:0022610 | biological adhesion | 108 | 960 | 2.95E-10 |
| GO:0007155 | cell adhesion | 108 | 960 | 2.95E-10 |
| GO:0048666 | neuron development | 30 | 154 | 3.69E-10 |
| GO:0042995 | cell projection | 48 | 313 | 3.87E-10 |
| GO:0048518 | positive regulation of biological process | 116 | 1062 | 4.78E-10 |
| GO:0000287 | magnesium ion binding | 61 | 447 | 7.84E-10 |

^*^A *P*-value is calculated representing the probability that the observed numbers of counts could have resulted from randomly distributing this GO term between the tested group and the reference group [25].
